# Clinical Research on Positron Emission Tomography Imaging of the Neuro-Stimulation System in Patients with Cochleo-Vestibular Implants: Is There a Response Beyond the Peripheral Organ?

**DOI:** 10.3390/jcm14051445

**Published:** 2025-02-21

**Authors:** Joan Lorente-Piera, Elena Prieto, Ángel Ramos de Miguel, Manuel Manrique, Nicolás Pérez-Fernández, Ángel Ramos Macías, Jaime Monedero Afonso, Alina Sanfiel Delgado, Jorge Miranda Ramos, Paula Alonso Alonso, Javier Arbizu, Raquel Manrique-Huarte

**Affiliations:** 1 Department of Otorhinolaryngology, Clínica Universidad de Navarra, 31008 Pamplona, Spain; mmanrique@unav.es (M.M.); rmanrique@unav.es (R.M.-H.); 2 Medical Phyics Department, Clínica Universidad de Navarra, 31008 Pamplona, Spain; eprietoaz@unav.es; 3 University Institute of Intelligent Systems and Numeric Applications, Complejo Hospitalario Universitario Insular MaternoInfantil, 35017 Las Palmas, Spain; aramosdm@fciisc.es; 4 Department of Otorhinolaryngology, Clínica Universidad de Navarra, 28027 Madrid, Spain; nperezfer@unav.es; 5 Department of Otorhinolaryngology, Complejo Hospitalario Universitario Insular MaternoInfantil, 35016 Las Palmas, Spain; ramosorl@idecnet.com (Á.R.M.); jaimemonedero1991@gmail.com (J.M.A.); 6 Department of Nuclear Medicine, Complejo Hospitalario Universitario Insular MaternoInfantil, 35016 Las Palmas, Spain; asandelj@gobiernodecanarias.org (A.S.D.); amirram@gobiernodecanarias.org (J.M.R.); 7 School of Medicine, Universidad de Navarra, 31009 Pamplona, Spain; palonsoalon@alumni.unav.es; 8 Department of Nuclear Medicine, Clínica Universidad de Navarra, 31008 Pamplona, Spain; jarbizu@unav.es

**Keywords:** vestibular implant, cochlear implant, PET, vertigo, hearing loss

## Abstract

**Introduction:** In patients refractory to vestibular rehabilitation in the management of bilateral vestibulopathy, the cochleo-vestibular implant has emerged as a viable alternative to enhance both audiovestibular function and quality of life. The main objective of this study is to pioneer the use of PET to assess cortical modifications in patients with cochleo-vestibular implants, aiming to evaluate the safety and functional improvements in individuals with bilateral vestibulopathy and severe to profound hearing loss. **Methods:** A phase I pilot clinical trial was conducted with participants who received a BIONIC-VEST CI24RE cochleo-vestibular implant, with pre- and post-implantation assessments conducted for twelve months. Audiovestibular testing and two PET studies with 18F-FDG under baseline conditions and with active stimulus to observe cortical-level differences were performed. **Results:** Five patients were included in the study, all of them treated with a cochleo-vestibular implant, none of whom presented postoperative adverse effects. Audiologically, the mean post-implant gain was 56.63 ± 14.53 dB and 50.40 ± 35.54% in terms of speech intelligibility. From a vestibular perspective, the most remarkable findings were observed at the graviceptive pathway level, where a mean posturographic improvement was observed, with a sensory organization test score of 24.20 ± 13.74 and a subjective visual vertical of 1.57° ± 0.79°, achieving, in most cases, results within the normal range (<2.3°) by the end of the follow-up. PET images confirmed that with the electrical stimulus active (implant ON), there was a supratentorial activation pattern, particularly in areas related to somatosensory integration, emotional regulation, and autonomic control. **Conclusions:** The BIONIC-VEST implant significantly improved the vestibular system, particularly the graviceptive pathway, enhancing balance and SVV and reducing fall risk. PET revealed distinct uptake patterns in baseline and activated conditions, highlighting a cortical-level response with the use of the cochleo-vestibular implant.

## 1. Introduction

Bilateral vestibulopathy is a chronic condition characterized by atypical clinical presentations, multiple forms of manifestation, and diverse therapeutic approaches aimed at mitigating its consequences. It is a rare condition, with a prevalence of 28 cases per 100.000 individuals in the United States in 2017 [1], caused by a bilateral deficit in vestibular reflexes. Notably, asymmetry in the information from both vestibular receptors does not always occur, which explains why typical symptoms such as nystagmus, vegetative symptoms, or rotational vertigo are absent in some cases. Instead, it often presents with nonspecific symptoms, including reduced visual acuity or a strong aversion to oscillatory body movements while walking (oscillopsia), stemming from the inability to stabilize the visual environment during head movements, leading to measurable alterations in the VOR. In 2017, diagnostic criteria for this condition were established by Strupp et al. [1].

Bilateral vestibular dysfunction profoundly impacts patients’ quality of life. The inability to perform daily activities normally leads to significant reductions in mobility and independence. Furthermore, this condition often results in a persistent fear of falling, which can lead to reduced physical activity and, ultimately, social isolation. Reduced activity not only exacerbates muscle weakness but also contributes to psychological and cognitive complications such as depression and anxiety [2].

Although bilateral vestibulopathy is a chronic and potentially disabling condition, patients can benefit from therapeutic interventions that promote central compensation and adaptation. Vestibular rehabilitation, both pharmacological and behavioral, focuses on stimulating central pathways involved in balance processing to make more efficient use of visual and somatosensory signals, compensating for the loss of vestibular function. Specific exercises designed to improve postural stability, coordination, and sensory adaptation can help patients enhance their mobility and reduce the risk of falls [3,4]. According to the literature, the success rate of these exercises in bilateral vestibulopathy is approximately 50% [5,6].

Despite the advances and generally positive outcomes achieved with vestibular rehabilitation, it is important to recognize that while this strategy can improve quality of life, the complete recovery of vestibular function is not possible in most cases of bilateral vestibulopathy. For this reason, emerging technologies, such as cochleo-vestibular implants, have been explored over the past decade as potential solutions to restore some of the lost audiovestibular functions [7].

Although various cochleo-vestibular implant models currently exist, including semicircular canal implants [8,9] and multichannel models [10,11], this study will primarily focus on implants targeting the otolithic organ. Their main objective is to restore the perception of gravity and linear accelerations [12]. The otolithic organs, including the utricle and saccule, are responsible for detecting head tilt and linear accelerations, such as those experienced when the body moves forward or backward. Dysfunction in these organs can cause severe balance problems, particularly in the perception of body position in space [13]. This could be explained by the fact that the stimulation of the otolithic organs may help to restore the perception of body position in space and improve postural stability, which is essential for preventing falls and enhancing mobility in patients with vestibular deficiency.

On the other hand, due to the proximity of auditory and vestibular structures, it has been hypothesized that stimulation with a cochlear implant (CI) may occasionally activate vestibular nerve afferents [14,15,16]. Recent studies have demonstrated that unintended interference can occur during electrical stimulation with the cochlear implant, with reports of patients experiencing vestibular symptoms during CI activation [17,18,19,20]. It has been suggested that these symptoms may be mediated by vestibular co-stimulation due to a phenomenon known as excitation spread, where CI currents propagate to surrounding neural tissues and structures.

Considering our group’s previous positive experiences in studying the auditory pathway in candidates for cochlear implantation with PET [21,22], we consider the need for these kinds of neuroimaging studies in patients with bilateral vestibulopathy for two main reasons. First, neuroimaging has contributed to a better understanding of vestibular disorders by providing a direct window to observe the functional and structural brain alterations underlying these conditions. Second, another rationale for exploring functional neuroimaging studies involves phenomena related to electrical vestibular stimulation. This has been studied in the past, primarily to investigate the anatomical and neurophysiological structures and pathways of the vestibular system. Cohen and Suzuki indicated, after studying animals, that eye movements could be elicited by electrically stimulating the ampullary and otolithic nerves [23]. An important finding was the evidence of convergence between ampullary and otolithic inputs on individual vestibular neurons.

Finally, neuroimaging has proven to be a valuable tool in the differential diagnosis of vestibular disorders, particularly in distinguishing between central and peripheral vertigo, which is critical for selecting appropriate treatment. Techniques like PET and SPECT have identified specific activation patterns in the brainstem and temporal cortex in cases of central vertigo that are absent in peripheral vertigo [24]. Although this study is based on the use of PET, it is important to remember that functional magnetic resonance imaging has also revealed alterations in gray matter and functional connectivity in patients with chronic vertigo, providing insights into the underlying neurobiological mechanisms [25,26]. These advancements not only enhance diagnostic precision but also support personalized therapeutic approaches such as targeted vestibular rehabilitation and cognitive behavioral therapy, which have been effective in alleviating symptoms in affected patients.

This study aims to become a pioneering model in the research of implantable devices for vestibular dysfunctions by focusing on the measurement and categorization of cortical modifications through PET in patients undergoing cochleo-vestibular implant placement. Additionally, it seeks to determine safety and functional improvement, as assessed through various audiovestibular tests, following implantation in patients with bilateral vestibulopathy and severe to profound bilateral hearing loss.

## 2. Materials and Methods

### 2.1. Study Design

A pilot study of a phase I multicenter clinical trial was conducted between 2018 and 2024 across two tertiary hospitals: Clínica Universidad de Navarra, Pamplona, Spain, and C.H.U. Insular Materno-Infantil, Las Palmas, Spain. The resulting design was a prospective within-subject repeated-measures study with a 12-month follow-up period.

This study adhered to the principles outlined in the 1964 Helsinki Declaration, including its subsequent revisions or equivalent ethical guidelines. Written informed consent was obtained from all participants prior to their inclusion in the study. All procedures involving human subjects complied with the ethical standards set by our institutional research committee. The study protocol was reviewed in January 2018 and received approval from the Ethics Committee of Clínica Universidad de Navarra under the approval number CEI 2020.104 and was funded by the European Union’s Horizon 2020 Research and Innovation Programme under Grant Agreement No. 801127.

**Figure 1:**
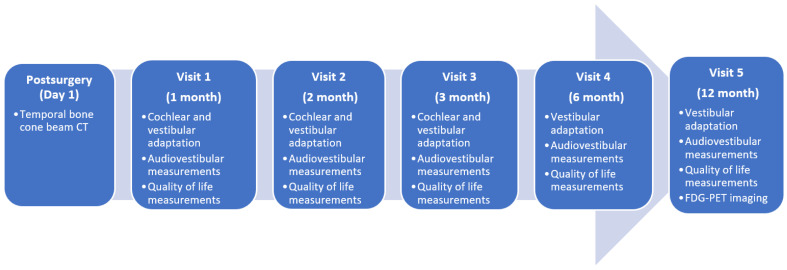
Summary of the postoperative follow-up conducted on the patients included in the study.

Evaluations prior to surgery were conducted within the two months leading up to implantation, while postoperative assessments were scheduled at follow-up intervals of one, two, three, six, and nine to twelve months. Testing took place under two specific conditions: when the implant was turned OFF, with no stimulation of the cochlear and vestibular systems, and when it was turned ON, providing simultaneous stimulation to both the cochlear and saccular systems. The study design, along with the post-implantation follow-up schedule, is summarized in Figure 1.

### 2.2. Cochleo-Vestibular Implant Patient Selection: Inclusion and Exclusion Criteria and the Implant Selected

Participants in the clinical trial must meet the Bárány Society’s criteria for bilateral vestibulopathy (BVP), which include a VOR gain of less than 0.6 in both ears, as well as severe to profound bilateral hearing loss qualifying them for cochlear implantation. Eligibility is restricted to individuals over 18 years of age who have experienced persistent vestibular symptoms for over a year without improvement and have no anticipated recovery. Imaging must confirm suitable inner ear anatomy, and candidates must demonstrate absent or difficult-to-elicit cVEMP and oVEMP responses in the targeted ear.

Exclusion criteria encompass the inability to provide informed consent, normal vestibular function, unilateral or compensated vestibular loss, and central or cerebellar dysfunction (e.g., CANVAS syndrome). Additional exclusions apply to those with persistent postural-perceptual dizziness (PPPD), mild to moderate hearing loss, inner ear malformations that hinder full electrode insertion, retrocochlear or central auditory dysfunction, and psychiatric or medical conditions contraindicating surgery. Individuals with unrealistic expectations, substance abuse issues, or use of vestibular suppressants are also excluded. Furthermore, conditions such as orthostatic tremor, oculomotor dysfunction, peripheral neuropathies, atypical parkinsonian syndromes, multisystem atrophy, and other central gait disorders disqualify potential participants.

When referring to the implant used in this study, we utilized the BIONIC-VEST CI24RE model (Cochlear, Sidney, Australia), which comprises two main components: an internal and an external part. The internal component includes a coil, an intracochlear electrode array with 19 contacts configured in the CI512 electrode array design, a vestibular electrode array with three full-band contacts spaced 0.2 mm apart, and a reference ball electrode. The external component corresponds to the commercially available Nucleus^®^ 6 or 7 sound processor, manufactured by Cochlear^®^ in New South Wales, Australia.

### 2.3. Examination and Complementary Tests

All patients underwent a physical examination, including otoscopy and an otoneurological evaluation using a videonystagmography (VNG) system (VideoFrenzel Interacoustics VF505m, Assens, Denmark). Audiovestibular assessments included pure-tone audiometry (PTA) (AC40, Interacoustics), vestibular evoked myogenic potentials (VEMPs, Eclipse Interacoustics, Assens, Denmark), the video head impulse test (vHIT, ICS Impulse GN Otometrics^®^ Natus Medical, Taastrup, Denmark), dynamic posturography (Equi-Test, NeuroCom Internacional, Inc., Clackmas, OR, USA), and subjective visual vertical (SVV, Difra Verti Equipment, Sidney, Australia). For imaging studies, all patients underwent magnetic resonance imaging (MRI, MAGNETOM Skyra, Siemens, Erlangen, Germany) and computed tomography scans (Biograph Vision™ 450 Siemens Medical Solutions, Erlangen, Germany).

These tests were conducted at the time of diagnosis, with VEMPs, PTAs, and vHIT repeated during follow-up.

- Audiological Evaluation: Findings were reported as pure-tone averages (PTAs), including pre- and post-treatment PTAs and gains, measured in decibels (dB) at frequencies of 500, 1000, 2000, 3000, and 6000 Hz. The degree of hearing loss was classified according to the criteria of the Bureau International d’Audiophonologie (BIAP) [27].
- Vestibular Evaluation: The vHIT was used to analyze the vestibulo-ocular reflex (VOR) gain, with values below 0.8 considered abnormal, and to evaluate the presence of corrective saccades in all three semicircular canals in both ears [28]. For VEMPs, both cervical (cVEMP) and ocular (oVEMP) tests were conducted. Abnormal vestibular function was defined as a VEMP response with an interaural asymmetry ratio (IAAR %) exceeding 40%. Asymmetry ratios were analyzed for air-conducted stimulation at 0.5 kHz, 1 kHz, and 4 kHz, with the acoustic stimulus intensity set at 97 dB normalized [29]. For the dynamic posturography study, following the principles outlined by Cevette et al. in 1995 [30], five distinct ratios were analyzed: vestibular, somatosensory, visual, visual preference, and overall result. For the SVV, the normal deviation range, defined as within 2.3° from the true vertical, was used as a reference [31].
- Imaging Studies: Thin-section helical CT scans with a slice thickness of 0.4 mm were used to assess the integrity of cochlear and vestibular structures, which could influence the surgical approach. These scans were performed both prior to surgery and one day after the procedure to ensure the correct positioning of the electrodes. Additionally, an MRI was performed exclusively before the surgery to evaluate the integrity of the previously described structures as well as the vestibulocochlear nerves.

### 2.4. PET Protocol in Patients with Cochleo-Vestibular Implants

Two patients were acquired from Clínica Universidad de Navarra, Pamplona, and three from C.H.U. Insular Materno-Infantil, Las Palmas, Spain. PET scans were performed using a Siemens Biograph Vision PET/CT scanner at both sites. Two brain PET scans were conducted within a 24 h interval under two different conditions in each patient: (1) With the implant in ON mode. (2) With the implant in OFF mode (deactivated two hours prior to imaging to prevent residual stimulation effects). It is important to note that a controlled environment was maintained between the phases. Images were reconstructed to a 440 × 440 matrix with the usual parameters for brain PET at each site.

PET scans were spatially normalized into a Montreal Neurological Institute (MNI)-based PET template and smoothed using a three-dimensional Gaussian kernel with SPM12 software 9.14 (Wellcome Trust Centre for Neuroimaging, UCL, London, UK). Individual FDG uptake values were normalized by proportional scaling to the global mean of the whole brain.

Normalized PET images in the OFF and ON conditions were subtracted to create a parametric map, where the value for each voxel represented the change in brain activity. The subtraction map was thresholded to display only voxels with statistically significant differences in brain activity between conditions (2 standard deviations) using a custom Matlab tool (Nodule 4-2).

The study subjects were evaluated both at rest and during cochleo-vestibular stimulation in both ears, using the activation of the BIONIC-VEST cochleo-vestibular implant as the sole stimulus. In both scenarios, the tests were conducted in silence and darkness.

**Figure 2:**
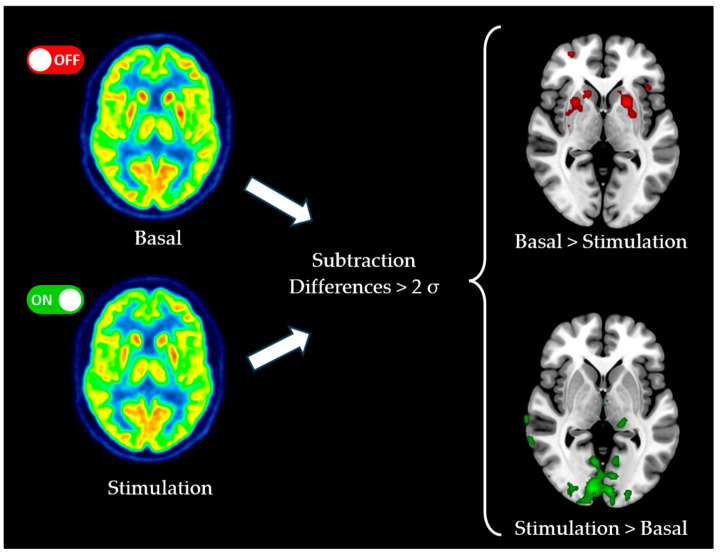
Summary of the PET subtraction algorithm applied in our study. Subtraction maps are presented over a standard MRI image.

To visualize the data, Surfice.exe software 6.0 (Columbia, SC, USA) was used. The cortical areas where the FDG uptake decreased when the implant was activated with respect to baseline conditions were displayed in red (uptake with implant OFF > uptake with implant ON). Conversely, the areas with increased FDG uptake during stimulation were presented in green (uptake with implant OFF < uptake with implant ON). The applied algorithm is summarized in Figure 2.

### 2.5. Surgical Procedures

For the cochleo-vestibular BIONIC-VEST implant placement, a retroauricular approach was also employed, with an extended posterior tympanotomy to ensure the correct placement of the electrodes in the cochlea through a round window approach, while the vestibular electrodes were positioned near the inferior vestibular nerve in the saccular region through a 0.5 mm stapedotomy using a CO_2_ laser (AcuPulse DUO, Lumenis Ltd., Yoneam, Israel).

The reference electrode is conventionally positioned beneath the periosteum, oriented toward the zygomatic arch. Stimulation parameters involve generating balanced biphasic pulse trains. The pulse width is set to 25 µs, with a stimulation frequency of 900 pulses per second. Monopolar stimulation is applied, utilizing the ball and plate electrodes as the reference (MP1+2). The modulation of stimulation varies depending on the target structure. For cochlear stimulation, the amplitude of the stimulus is modulated between the threshold level (T) and the comfort level (C), defining the dynamic range. In contrast, otolith stimulation is delivered at a constant current level.

### 2.6. Cortical Areas Under Study in Neuroimaging Tests with FDG-PET

According to the existing literature in this field [32,33,34,35], the areas studied were organized into functional families.

**Figure 3:**
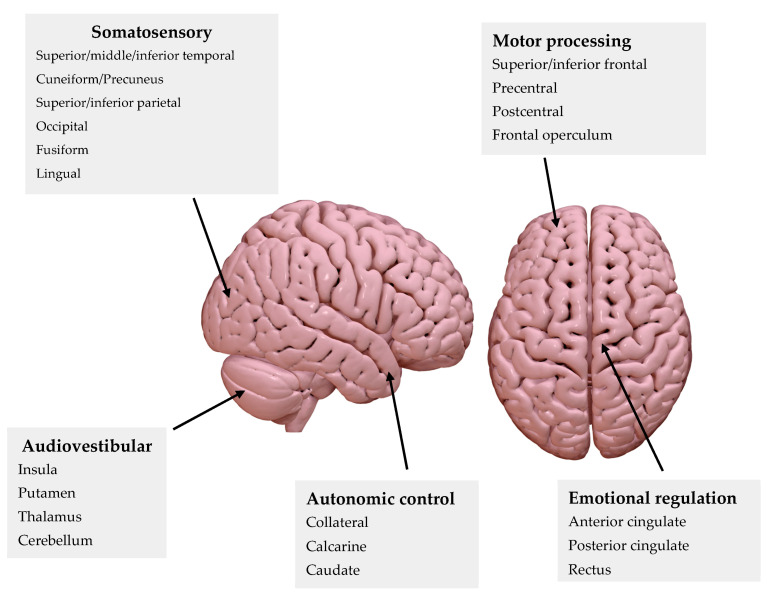
Cortical representation and summary of the different areas studied in the clinical trial using PET-CT imaging.

We divided the cortical areas under study, from a functional perspective, into five distinct subgroups: audiovestibular processing, motor processing, somatosensory integration and processing, emotional regulation, and autonomic control. This distribution is represented in Figure 3.

### 2.7. Statistical Analysis

A comparative study of paired samples was proposed (audiovestibular outcomes before and after treatment; PET scan results before and after treatment) by comparing means using the Student’s *t*-test or medians with the sign test or Wilcoxon test, depending on whether normality criteria were met. Normality criteria were assessed using the Shapiro–Wilk test and visual comparisons. A *p*-value < 0.05 was considered statistically significant. Additionally, 95% confidence intervals (CIs) were calculated to estimate the range of the effects. Data collection and analysis were performed using RStudio version 4.3.3 (Boston, MA, USA).

## 3. Results

### 3.1. Population and Etiologies

The study initially included six participants diagnosed with BVP and severe to profound bilateral hearing loss. Unfortunately, one participant was unable to complete the study due to other comorbidities that prevented proper adherence to cochleo-vestibular device usage.

Thus, a total of five subjects were included in the pilot study. All of them were treated with the cochleo-vestibular implant, and all of them were male. The average age of all patients was 55.60 ± 13.89 years, and the mean disease duration was 19.61 ± 18.52 years.

When identifying the causes, we found that the etiology underlying the symptoms leading one patient in the cohort to seek specialist care remained unknown, although this individual had an underlying diagnosis of Parkinson’s disease. On the other hand, the most common causes were bilateral Ménière’s disease in two patients, the same proportion as post-meningitic sequelae.

It is important to note that only Subject 2 (with a left cochlear implant) and Subject 3 (with bilateral hearing aids) had received any type of auditory assistance prior to the placement of the cochleo-vestibular implant in the ipsilateral ear. These all findings are summarized in Table 1.

### 3.2. Hearing Evaluation

The statistical analysis of the mean pure-tone averages (PTAs) obtained through pure-tone audiometry in the five patients with cochleo-vestibular implants showed significant changes after the intervention. Pre-implantation, the mean PTA was 95.30 ± 16.38 dB, which significantly decreased to 41.90 ± 26.16 dB post-implantation, resulting in an average gain of 53.40 ± 12.54 dB (95% CI: 44.99 to 65.81 dB). This change was statistically significant, as evidenced by the statistical analysis (t = 14.77; *p* < 0.0001).

**Figure 4:**
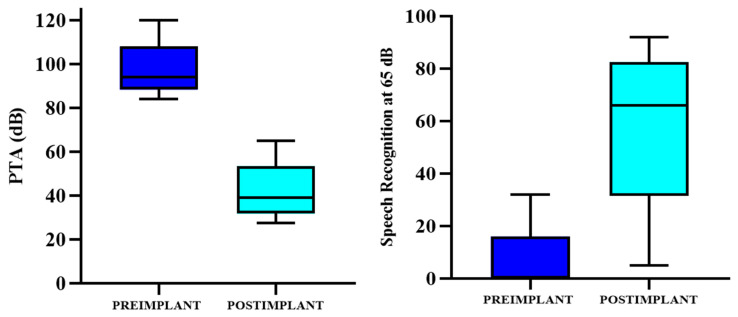
Progression of auditory performance recorded in the PTA (**left** panel) and rate of discrimination (**right** panel) in the ipsilateral ears of each patient included in the study.

The statistical analysis of speech discrimination at 65 dB, measured through speech audiometry in the five patients with cochleo-vestibular implants, revealed a pre-implantation discrimination rate with a mean of 8.40 ± 18.78%. Post-implantation, this significantly increased to 58.80 ± 32.60%, resulting in an average gain of 50.40 ± 35.54% (95% CI: 44.99–65.81 dB). This change was statistically significant (t = −3.45; *p* = 0.0260). Both situations are illustrated in Table 2 and Figure 4.

### 3.3. Vestibular Evaluation

The statistical analysis of the results obtained using the vHIT in the lateral (LSC), posterior (PSC), and superior (SSC) semicircular canals of patients with cochleo-vestibular implants turned on revealed no statistically significant differences in VOR gains when comparing either pre- and post-implantation or ipsilateral and contralateral sides.

In the horizontal semicircular canal (LSC), the pre- and post-implantation values on the ipsilateral side showed a gain of 0.02 ± 0.22 (95% CI: −0.25 to 0.29), with no significant differences (t = −0.20; *p* = 0.852); the results were similar on the contralateral side, with a gain of 0.01 ± 0.25 (95% CI: −0.30 to 0.32; t = 1.49; *p* = 0.210). Additionally, when comparing gains between the two sides, no significant differences were found (t = 0.86; *p* = 0.437).

In the posterior semicircular canal (PSC), no significant differences were observed when comparing pre- and post-implantation values either on the ipsilateral side, with a gain of 0.02 ± 0.23 (95% CI: −0.27 to 0.31; t = −0.24; *p* = 0.825), or on the contralateral side, with a gain of 0.02 ± 0.18 (95% CI: −0.20 to 0.24; t = 0.22; *p* = 0.833). Similarly, the gains between ipsilateral and contralateral sides did not show significant variation (t = 0.48; *p* = 0.657).

**Figure 5:**
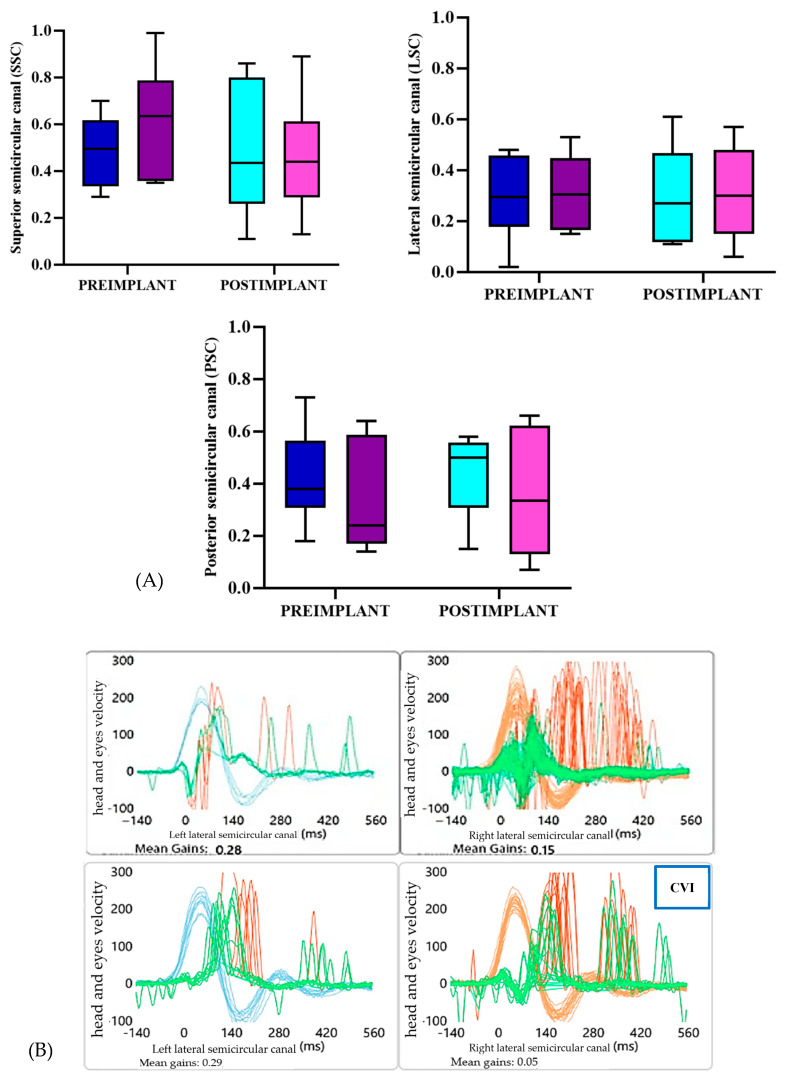

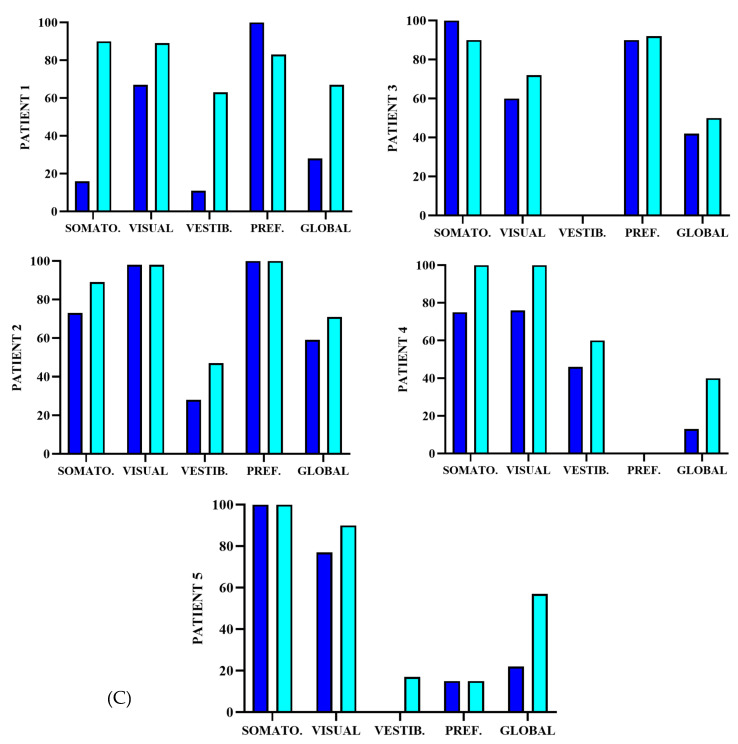
(**A**). Summary of the different gains recorded in the vHIT in patients from the trial, analyzing the three different canals separately. The blue colors represent the ear ipsilateral to the cochleo-vestibular implant, while the purple colors represent the contralateral ears. (**B**). Example of vHIT results for the lateral semicircular canals of one of the patients in the trial. It shows two scenarios: the top image corresponds to the pre-implantation phase, and the bottom image shows the post-implantation phase with the cochleo-vestibular implant (CVI). Despite the lack of improvement in gain, clear refixation saccade phenomena can be observed. (**C**). Representation of the evolution of the different quotients included in the SOT. The dark blue color corresponds to the pre-implantation moment and the light blue color corresponds to post-implantation. SOMATO: somatosensorial; VESTIB: vestibular; PREF: visual preference.

In the superior semicircular canal (SSC), the pre- and post-implantation values on the ipsilateral side also showed no significant differences, with a decline of 0.05 ± 0.15 (95% CI: −0.24 to 0.14; t = 0.80; *p* = 0.468). The same was observed on the contralateral side, with a change of −0.17 ± 0.19 (95% CI: −0.41 to 0.07; t = −0.56; *p* = 0.603). The comparison of gains between the ipsilateral and contralateral sides also revealed no significant differences (t = −1.06; *p* = 0.348). These changes can be observed in Figure 5A. 

Another particularly relevant finding we observed in the vHIT, despite the gains not showing improvement with the implant, was the reorganization of saccades. Before implantation, all five patients exhibited both covert and overt refixation saccades, which were disorganized. This changed after the placement of the implant. In other words, despite the persistence of both types of saccades, they showed a clear tendency toward organization, as shown in Figure 5B.

Both before and after the placement of the cochleo-vestibular implant, all patients demonstrated an absence of response in the VEMPs, indicating a lack of vestibular myogenic function or vestibular areflexia on both the ipsilateral and contralateral sides.

When analyzing the graviceptive pathway of balance, arguably one of the most significant and impactful systems due to its role in transmitting stimuli through the otolithic organ, we primarily focused on the results of dynamic posturography and the subjective vertical visual (SVV).

Initially, the posturography results revealed a clear shift in postural patterns in the subjects with the BIONIC-VEST implant. In the first group, the analysis of the results obtained from the mean value of the sensory organization test (SOT) showed a pre-implantation mean of 32.80 ± 18.05 (95% CI: 10.39 to 55.21), which increased post-implantation to 57.00 ± 12.59 (95% CI: 41.37 to 72.63). This represents an average performance gain of 24.20 ± 13.74 (95% CI: 7.14 to 41.26). The *t*-test yielded a value of t = −3.94, *p* = 0.017, indicating that the observed improvement following implantation was statistically significant. These results are illustrated in Figure 5C.

When comparing the statistical significance of the various SOT quotients in posturography, we found that the results were statistically significant only for the global score (t = −3.94, *p* = 0.017) and the visual condition (t = −2.99, *p* = 0.040). In contrast, the somatosensory quotient (t = −1.45, *p* = 0.220), vestibular quotient (t = −2.31, *p* = 0.082), and visual preference quotient (t = −1.24, *p* = 0.284) did not yield statistically significant results. However, a highly relevant finding was that fall risk disappeared in the subjects with Ménière’s disease (Subjects 1 and 2) and in the subject with Parkinson’s disease of unknown etiology (Subject 3). Conversely, fall risk persisted in the two subjects with meningitis sequelae (Subjects 4 and 5).

**Figure 6:**
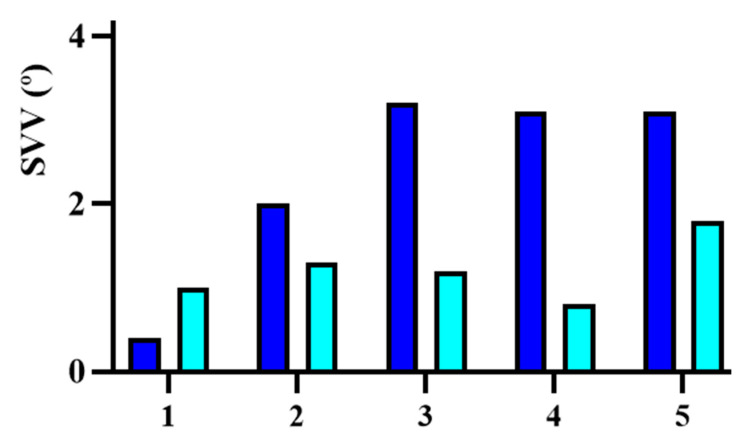
Representation of the evolution of SVV in the different subjects. The dark blue color corresponds to the pre-implantation moment and the light blue color corresponds to post-implantation.

Finally, in the vestibular section, an analysis of the SVV was also included. Before treatment, SVV values varied widely among patients, indicating a distorted perception of verticality. The mean pre-treatment SVV value was 4.00° ± 3.14° (95% CI: 0.10° to 7.90°), as shown by the blue bars in Figure 6. After the implant was placed, SVV values showed a significant improvement in most patients, with a mean post-treatment value of 1.57° ± 0.79° (95% CI: 0.59° to 2.55°), represented by the cyan bars in the same figure. The analysis revealed an average improvement of 1.86° ± 0.89° (95% CI: 0.75° to 2.97°) following implantation. Statistical testing demonstrated significant differences between pre- and post-implantation values (t = 4.67, *p* = 0.009), confirming that the observed improvement after treatment was statistically significant. It is important to highlight that only the subjects with the cochleo-vestibular implant achieved values within the normal range (<2.3°).

As can be inferred, the results are represented individually for each patient in the different SOT and SVV ratios rather than collectively. This decision is based on the importance of conducting a thorough analysis of the tests that measure the graviceptive pathway of balance, considering that the implant used directly stimulates the otolithic organ.

### 3.4. PET Results

When analyzing PET images based on the different areas classified in Section 2.6, we observed a tendency toward a decrease in uptake in the audiovestibular and motor processing areas in all patients, except for Subject 2, who paradoxically exhibited the opposite behavior, with an increase in uptake with the stimulus present. However, slight divergences were observed in these areas, as seen at the cerebellar level in Subjects 1 and 3, where there was greater uptake with the BIONIC-VEST activated, or at the putamen, which showed a similar pattern but in this case in subjects with post-meningitic sequelae (Subjects 4 and 5).

On the other hand, when analyzing areas related to sensory integration and visuospatial perception, as well as emotional regulation and autonomic control, we observed the opposite trend, with a generalized increase in uptake with the implant activated, except in the superior temporal area, which predominantly showed a decrease in uptake in all patients, except again for Subject 2. However, in these areas, there are also other peculiarities, especially in Subject 2, who exhibited a decrease in uptake when the implant was off in areas such as the rectus gyrus, collateral gyrus, and calcarine gyrus, which are primarily involved in emotional regulation and autonomic control. This was the opposite of the other four cases, which showed a clear increase in patterns of uptake in these regions.

**Figure 7:**
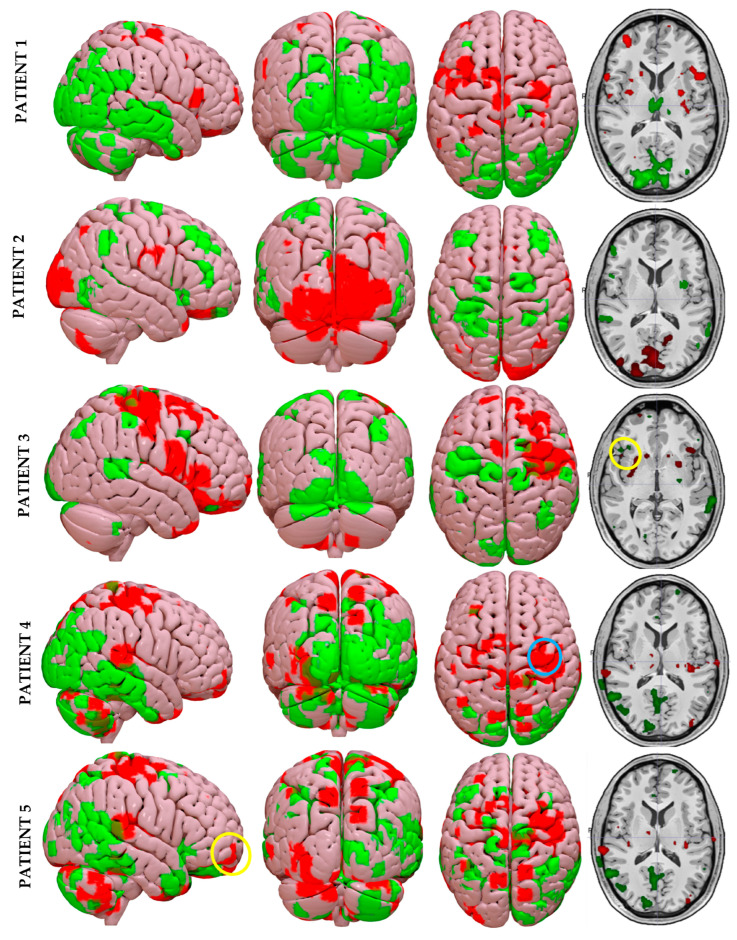
Subtraction PET images illustrating the metabolic changes between baseline conditions and electrical stimulation. The yellow circle in patient 3 represents notable uptake at the ipsilateral frontal operculum related to the implant, while the blue circle in the fourth patient shows a decrease in uptake in the ipsilateral precentral gyrus, and the yellow circle in the fifth subject indicates an increase in uptake with the presence of electrical stimulation in the ipsilateral inferior frontal gyrus.

Regarding the specific findings highlighted in Figure 7, we can observe that in Subject 3, who has an unknown peripheral etiology and underlying Parkinson’s disease, the previously described trend is followed, with notable uptake at the ipsilateral frontal operculum related to the implant (yellow circle), a cortical area responsible for regulating facial tone and expression, a function that is compromised in patients with parkinsonism. Subjects 4 and 5, who have post-meningitic sequelae, exhibit a very similar uptake pattern that aligns with the general trend, with particularly notable heterogeneity at the occipital and cerebellar levels, especially in Subject 5, who exhibits an increase in metabolism with the stimulus present ipsilaterally but a decrease in uptake contralaterally. However, the higher uptake in baseline conditions is mainly observed in motor areas such as the precentral gyrus (blue circle) and the inferior frontal gyrus (yellow circle).

## 4. Discussion

The hypothesis underpinning this study is that otolith stimulation via vestibular implants may play a key role in improving stability and quality of life, given the critical role of the otoliths in maintaining balance [36], in patients with bilateral vestibulopathy for whom vestibular rehabilitation does not provide sufficiently satisfactory results for them to carry out their daily activities. We confirmed that otolithic implants, which primarily stimulate the inferior branch of the vestibular nerve (mainly innerving the saccule), aim to restore vestibular function, significantly enhancing daily functionality [37]. Moreover, the surgical procedure for device placement, following the standards of minimally traumatic surgery, did not induce adverse effects in our subjects, thus meeting the safety requirements inherent to phase I of a clinical trial, as is the context of this study.

Diagnosing the etiology of bilateral vestibulopathy remains a challenge, even after its identification. Previous studies, such as those by Rinne et al. [38], Lucieer et al. [39], and Moyaert et al. [40], have shown that in 49–80% of cases, a probable or definitive etiology can be determined, leaving 20–51% of cases as idiopathic. The etiologies for bilateral vestibulopathy are diverse, and the underlying causes of idiopathic cases remain debated. Reflecting this variability, our study cohort of five patients with bilateral vestibulopathy and severe to profound hearing loss presents a heterogeneous distribution of causes: two cases followed acute events such as post-meningitic infection (one with additional contralateral trauma), one was of unknown progressive origin, and two were caused by Ménière’s disease.

To date, this is the first study that combines the outcomes of a cochleo-vestibular device and documents the supratentorial changes occurring after implantation using functional imaging techniques based on PET. Thus, the most innovative aspect of this study is the evaluation of five patients with severe audiovestibular deficits using functional neuroimaging. The results revealed distinct patterns depending on etiology, treatment in the contralateral ear, and disease progression. However, the general insight we can draw from this study is that an electrical stimulus applied through the BIONIC-VEST device induces changes in the vestibular pathway that go beyond the peripheral organ, thus also producing modifications at the central level as a result of a change in the metabolic pattern that arises from electrical stimulation with the implant. In other words, this study confirms that an otolithic cochleo-vestibular implant can generate a supratentorial response beyond the inner ear.

Among patients with Ménière’s disease, Subject 1 demonstrated greater uptake in the cerebellum with the implant activated, indicating improved sensorimotor integration. In contrast, Subject 2, who had a longer history of bimodal stimulation and half the disease duration, exhibited higher activation in the audiovestibular and motor areas. This supports the central compensation hypothesis described by Piccioti et al. [41] and Domènech-Vadillo [42]. These differences may also explain why the latter achieved the best overall functional outcomes in the cohort, particularly in postural stability and auditory performance.

In the basal ganglia, higher uptake with the implant off could indicate compensatory efforts in the absence of vestibular input, particularly in structures like the caudate nucleus and putamen, which are responsible for sensorimotor integration. In the case of the patient with idiopathic peripheral etiology and Parkinson’s disease, a marked decrease in uptake was observed in these areas, consistent with dopaminergic dysfunction and challenges in sensory integration, aligning with findings from Brosse et al. [43].

Patients with post-meningitic sequelae (Subjects 4 and 5) displayed homogeneous deactivation or decreased uptake with the implant off. This finding was seen specially in the motor and audiovestibular areas, except in the cerebellum, reflecting motor compensatory dependency, as can be inferred from Figure 7, with a striking and consistent higher uptake in baseline conditions in both subjects in areas such as the insula, superior and inferior frontal gyrus, or precentral and postcentral gyrus. When the implant was activated, increased uptake dominated in areas such as the putamen, underscoring its role in sensorimotor reorganization, as suggested by Muehlberg et al. [44]. In their 2024 study, this group demonstrated that dopamine availability in the sensorimotor putamen significantly influences motor learning. Furthermore, these subjects showed the highest uptake intensity in areas related to sensory integration, visuospatial perception, and emotional regulation. This may, on the one hand, suggest that electrical stimulation enhances sensory input, facilitating spatial perception and postural stability, or, on the other, emphasizes the importance of cortical and subcortical reorganization in long-term functional adaptation. Although the observed PET uptake differences suggest metabolic and functional reorganization beyond the peripheral vestibular structures, future studies incorporating complementary neuroimaging modalities (e.g., fMRI or perfusion imaging) will be necessary to further validate these findings.

Regarding audiovestibular functional outcomes, vestibular assessments included pre- and post-implantation vHIT testing, which showed no significant improvement in the six semicircular canals post-implantation, a statement that must be taken with caution since the results did not reach statistical significance. However, the reorganization of covert and overt refixation saccades, as shown in Figure 5B, was observed in all five patients with the BIONIC-VEST device, aligning with compensatory mechanisms described by Rey-Martínez et al. [45]. The vestibular compensation process is based on three fundamental pillars: restoration, habituation, and adaptation. Restoration involves recovering lost functional capacities and regenerating neuronal synapses damaged by injury, a phenomenon demonstrated in vivo thanks to neuronal plasticity [46,47]. On the other hand, habituation focuses on reducing vestibular asymmetry, while adaptation aims to utilize other sensory inputs, such as visual or somatosensory information, to compensate for the lack of vestibular stimulus [45,46].

Another interesting aspect is the discussion of the results of VEMPs, which were absent both in the preoperative and postoperative evaluations in all the five cases. This finding was also expected, given that although the BIONIC-VEST implant allows for the direct electrical stimulation of the inferior vestibular nerve via the saccule, both otolithic organs remained impaired, and thus, the presence of responses in cVEMPs or oVEMPs was not anticipated. To assess the efficacy at the otolithic level, the only methodology that would allow for direct confirmation would be the intraoperative recording of electrical VEMPs, as described in the work of Ramos-Macías et al. [48]. In this study, conducted by members of our group, electrical VEMPs were obtained using an electrical stimulus synchronized with specific monitoring systems. However, in the context of this study, electrical VEMPs were not performed in all patients, as this evaluation was not included in the BIONIC-VEST consortium protocol. Nevertheless, these results highlight the importance of incorporating such measurements in future studies to more accurately verify the efficacy of the implant at the otolithic level and its ability to activate specific reflexes related to the saccule and utricle.

First, when analyzing the graviceptive pathways, dynamic posturography results revealed significant improvements, although, once again, these statements should be taken with caution since, while the differences in the global or composite score did reach statistical significance, only the visual condition had a statistically significant *p*-value. The global SOT showed a statistically significant gain of approximately 25 points in the composite score when comparing pre- and post-implantation results. Patterns varied by etiology and disease progression: Ménière’s patients showed vestibular and somatosensory deficits, with visual deficits linked to longer disease duration in one case; the idiopathic case with Parkinson’s disease showed a persistent fall risk despite improved visual conditions and stability limits; and post-meningitic cases demonstrated persistent vestibular deficits despite improvements in other parameters.

The SVV was also key in evaluating vestibular dysfunction and the response to the BIONIC-VEST implant, showing significant improvement in all five cases, with an average reduction of 2° in deviation from the true vertical. Patients with Ménière’s disease showed smaller pre-implantation deviations (≤2°), while Subjects 3, 4, and 5 exhibited pathological deviations (>3°). At 12 months post-implantation, all patients achieved normal values, notably Subject 5 from the post-meningitic group, who presented a final deviation of 1.80°.

One key advantage of otolithic stimulation is its ability to restore gravitational perception. Healthy otoliths continuously perceive gravity, providing essential information on head orientation; when compromised, this leads to poor balance and a higher risk of falls [49,50]. Electrical otolith and galvanic stimulation recreates this gravitational reference, enhancing orientation and posture, a statement supported by the positive results observed in vestibular tests such as the SVV and SOT, showing overall improvements in all five patients treated with this device.

Historically, it has been suspected that electrical stimulation of the auditory nerve via cochlear implantation might affect the adjacent vestibular nerve or hair cells [51]. This cross-interaction could influence the excitability of vestibular pathways in response to physiological stimuli [52]. Thus, following the marked improvement observed in dynamic posturography and SVV with the BIONIC-VEST implant, it would be interesting to include a similar sample, from a demographic perspective and with comparable clinical criteria, to enable equivalent comparisons between subjects with a cochleo-vestibular implant and those with a single cochlear implant or even, in the future, with a single vestibular implant.

### Limitations

The main limitation of this study is the small sample size, with only five patients included. This inherently reduces the statistical power of the analysis and limits the ability to extrapolate findings to a broader population. However, it is important to highlight that this study was designed as a phase I clinical trial, where the primary objective is not to establish generalizability but rather to conduct an initial evaluation of safety and preliminary treatment effects. In this context, the sample size is acceptable and consistent with similar early phase trials, as these studies primarily aim to obtain preliminary data to justify and guide larger-scale future research after demonstrating the feasibility of the device and its associated procedures.

Another relevant limitation is the lack of randomization, which could introduce selection bias and limit direct comparisons. Although the study attempted to minimize bias by using a standardized inclusion protocol, future studies should incorporate randomized trials with larger and more diverse cohorts to better assess the clinical significance of the cochleo-vestibular implant. Additionally, the variability in vestibular outcomes must be acknowledged. While some patients demonstrated clear functional improvements, others exhibited heterogeneous responses, particularly in some specific conditions in posturography. As we explained, this variability may stem from differences in compensatory mechanisms, disease etiology, or individual neuroplastic adaptation, factors that should be further explored in future studies. To partially address this limitation, we conducted stratified analyses based on etiology and disease duration and included an extensive audiovestibular assessment of more than 20 tests per patient, alongside quantitative PET evaluations.

Finally, we emphasize that the findings of this study should be considered preliminary, as their validation requires larger and more representative samples. Despite these limitations, this study fulfills its purpose as an initial exploration of the supratentorial response to the cochleo-vestibular implant and an evaluation of the feasibility of this treatment. The results provide a foundation for more comprehensive future investigations that will help refine the clinical application of this technology.

## 5. Conclusions

The BIONIC-VEST implant provided significant improvements in the patients’ vestibular systems, particularly in the measurement and evaluation of the gravitational pathway. Due to the absence of direct stimulation of the semicircular canals, the improvements were primarily observed in the graviceptive pathway of balance, as evidenced by the results of dynamic posturography and SVV, as well as a reduction in fall risk.

Overall, PET studies of the neurostimulator system revealed a differentiated uptake pattern under the two conditions studied: baseline and with the implant activated. In both cases, it was evident that the cochleo-vestibular device promotes a metabolic central effect when it turns on.

## Figures and Tables

**Table 1:** Summary of the population characteristics included in the study. BIO-VEST: BIONIC-VEST; TBI: traumatic brain injury.

Patient	Age	Gender	Disease Evolution	Etiology	BIO-VEST Implant	Contralateral Ear
Patient 1	63 years	Male	44 years	Bilateral Ménière disease	Right	Hearing aid
Patient 2	40 years	Male	27 years	Bilateral Ménière disease	Right	Cochlear implant
Patient 3	70 years	Male	25 years	Idiopathic	Left	Hearing aid
Patient 4	51 years	Male	1.5 years	Bilateral meningitis	Left	Cochlear implant
Patient 5	58 years	Male	6 months	Meningitis and TBI	Right	Cochlear implant

**Table 2:** Summary of the progression of auditory performance recorded in the PTA and rate of discrimination in speech audiometry.

Patient	PTA Pre-Implant	PTA Post-Implant	Speech A. Pre-Implant	Speech A. Post-Implant
Patient 1	120 dB	65 dB	0%	5%
Patient 2	92.50 dB	27.50 dB	0%	92%
Patient 3	84 dB	42 dB	32%	66%
Patient 4	94 dB	36 dB	0%	73%
Patient 5	96 dB	39 dB	0%	58%

## Data Availability

Data pertaining to this study can be shared upon request to the corresponding author.
